# Anti-ovarian cancer migration and toxicity characteristics of a platinum(IV) pro-drug with axial HDAC inhibitor ligands in zebrafish models

**DOI:** 10.1007/s10637-024-01479-3

**Published:** 2024-10-21

**Authors:** Salma Begum, Scheldon D. Irvin, Carol K. Cox, Zhouyang Huang, Justin J. Wilson, Jerry D. Monroe, Yann Gibert

**Affiliations:** 1 Department of Cell and Molecular Biology, University of Mississippi Medical Center, 2500 State Street, Jackson, MS 39216 USA; 2 Department of Chemistry and Chemical Biology, Cornell University, Ithaca, NY 14853- 1301 USA; 3 Department of Chemistry and Biochemistry, University of California Santa Barbara, Santa Barbara, CA 93106-9510 USA

**Keywords:** Zebrafish, Platinum(IV), HDAC inhibitor, Xenografts, Toxicity, Cisplatin efficacy

## Abstract

Ovarian cancer is the fifth leading cause of cancer related death in the United States. Cisplatin is a platinum-based anti-cancer drug used against ovarian cancer that enters malignant cells and then damages DNA causing cell death. Typically, ovarian cancer cells become resistant to cisplatin making it necessary to increase subsequent dosage, which usually leads to side-effects including irreversible damage to kidney and auditory system tissue. Ovarian cancer resistance is often associated with upregulation of histone deacetylase (HDAC) enzymes that cause DNA to adopt a closed configuration which reduces the ability of cisplatin to target and damage DNA. Compound B, a platinum(IV) complex with two axial phenylbutyrate (PBA) HDAC inhibitor ligands attached to a cisplatin core, can simultaneously inhibit HDAC activity and damage DNA causing decreased cancer cell viability in cisplatin-sensitive (A2780) and -resistant (A2780cis) ovarian cancer cell lines. However, compound B was not previously evaluated *in vivo*. As simultaneously inhibiting HDAC-mediated resistance with cisplatin treatment could potentiate the platinum drug’s effect, we first confirmed the anti-cancer effect of compound B in the A2780 and A2780cis cell lines using the 3-(4,5-dimethylthiazol-2-yl)-2,5-diphenyltetrazolium bromide spectrophotometric assay. Then, we used zebrafish embryo and transgenic animal models to comparatively analyze the effect of cisplatin, compound B, and controls on general organismal, auditory, and renal system toxicity, and cancer metastasis. We found that lower dosages of compound B (0.3 or 0.6 µM) than of cisplatin (2.0 µM) could cause similar or decreased levels of general, auditory, and renal tissue toxicity, and at 0.6 µM, compound B reduces cancer metastasis more than 2.0 µM cisplatin.

**Supplementary Information:**

The online version contains supplementary material available at 10.1007/s10637-024-01479-3.

## Introduction

The platinum(II) complex, *cis*-[Pt(NH_3_)_2_Cl_2_] (cisplatin), has been successfully used against several cancers including ovarian, testicular, bladder, breast, non-small cell lung cancer, head and neck, and glioblastomas [1]. Cisplatin’s anti-cancer activity is due to its ability to enter into the nuclei of cells where it binds to and damages DNA causing activation of cell death pathways [2]. However, cisplatin treatment typically activates resistance mechanisms which necessitates increased dosage in subsequent treatments leading to permanent damage to renal and auditory cells [3, 4]. Platinum(IV) complexes with a platinum(II) core attached to functionalized axial ligands that are released upon cell entry and which can target specific cancer mechanisms have been proposed as a next-generation of chemotherapeutics [5]. Recently, researchers have synthesized several platinum(IV) complexes derived from cisplatin with various histone deacetylase (HDAC) inhibitor axial ligands and found that these compounds can exhibit higher anti-cancer activity than the platinum(II) drug including in cisplatin-sensitive and -resistant ovarian cancer cell lines [6, 7]. This approach is premised on HDAC inhibition functioning to remove acetyl groups from histone and non-histone substrates leading to modified regulation of oncogenes and tumor suppressor genes and causing chromatin to adopt an open configuration making DNA more accessible to cisplatin binding [8–11]. In fact, cisplatin treatment in combination with HDAC inhibitors can potentiate the platinum compound’s anticancer activity by modulating cell cycle, cell growth, and apoptosis genes [12, 13].

Platinum(IV) pro-drugs combining cisplatin with phenylbutyrate (PBA) can produce synergistic effects that include greater drug potency in cisplatin resistant ovarian cancer cell lines [6]. This data suggests that platinum(IV) complexes with HDAC axial ligands might be advantageous if they can exert their effects against cisplatin resistant cancer at lower dosages that might avoid inducing side-effects. However, platinum(IV) complexes with HDAC inhibitor axial ligands have not yet been evaluated for their side-effect properties and ability to prevent cancer progression in animal models. Zebrafish models have been successfully used to assess the effect of drugs including platinum-based chemotherapeutic compounds on tissue toxicity and on cancer metastasis using xenografts [14–18]. Wild-type zebrafish have been used to assess the effect of cisplatin on general organismal toxicity using caspase-3 staining to measure apoptosis [19, 20]. Cisplatin induced nephrotoxicity has been assessed using structure-based (*wt1b*):eGFP transgenic models that express green fluorescent protein (GFP) in the zebrafish glomerulus and pronephric tubules [15] and fluorescent dye methods to measure renal function [14]. Further, the ototoxic effect of cisplatin can be measured using *brn3c* (*pouf4f3*):eGFP zebrafish transgenics that express GFP in neuromast hair cells [21]. Wild-type and transgenic zebrafish xenograft models are also routinely used with fluorescent cell staining techniques to measure how drugs affect cancer proliferation and metastasis by monitoring tumor area and markers of cell division and cell death [22–24].

In this project, we used cell culture methods and the 3-(4,5-dimethylthiazol-2-yl)-2,5-diphenyltetrazolium bromide (MTT) assay to measure the effects of control, cisplatin, and the platinum(IV) complex, *cis*,*cis*,*trans*-[Pt(NH_3_)_2_Cl_2_(PBA)_2_] (Compound B), which has a cisplatin-core with two PBA axial ligands [6], on the ovarian cancer cell lines, A2780 (cisplatin-sensitive) and A2780cis (cisplatin-resistant), that are widely used to assess metal complex chemotherapeutic toxicity [25–27]. Then, we microinjected A2780cis cells into wild-type zebrafish embryos, and monitored the metastasis of the cancer cells after exposing the embryos to the different treatments. As a final step, we used wild-type and transgenic zebrafish embryo models to assess the effects of control, cisplatin, and compound B treatments on general, nephro- and ototoxicity.

We found that compound B had lower IC_50_ values than cisplatin in both the A2780 and A2780cis cell lines. Further, we also found that both a lower and higher concentration of compound B (0.3 and 0.6 µM) caused a comparable reduction in cancer metastasis compared to cisplatin (2.0 µM) in zebrafish A2780cis cisplatin-resistant xenografts. In addition, compound B and cisplatin had similar general toxicity effects in AB zebrafish, but cisplatin caused reduced numbers of auditory hair cells. When we measured the effect of compound B and cisplatin on zebrafish pronephros area, there was no effect; however, in the renal function zebrafish assay, we found that only cisplatin treatment impaired filtration compared to control and compound B. This data suggests that a lower concentration of compound B than cisplatin may cause reduced auditory and renal tissue toxicity than cisplatin while retaining similar anti-ovarian cancer metastasis effect.

## Methods and materials

### Chemical synthesis materials and reagents

All reagents were purchased from commercial vendors. All solvents were of American Chemical Society grade or higher. 4-phenylbutanoic anhydride [28] and *cis*,* cis*,* trans*-[Pt(NH_3_)_2_Cl_2_(OH)_2_] [29] were synthesized as previously described.

### Physical measurements

Nuclear magnetic resonance (NMR) samples were prepared in DMSO-*d*6 and analyzed on a 400 MHz Bruker AV 3HD spectrometer with BBFO broadband probe. ^1^H NMR chemical shifts were referenced internally to tetramethylsilane (δ = 0 ppm). High-resolution electrospray mass spectrometry (HR-ESI-MS) measurements were obtained on an Exactive Orbitrap mass spectrometer in negative ion mode (ThermoFisher Scientific, Waltham, MA). Elemental analysis (CHN) was performed by Atlantic Microlab Inc. (Norcross, GA, USA).

### Synthesis of cis, cis, trans-[Pt(NH_3_)_2_CI_2_(PBA)_2_]

**Fig. 1:**
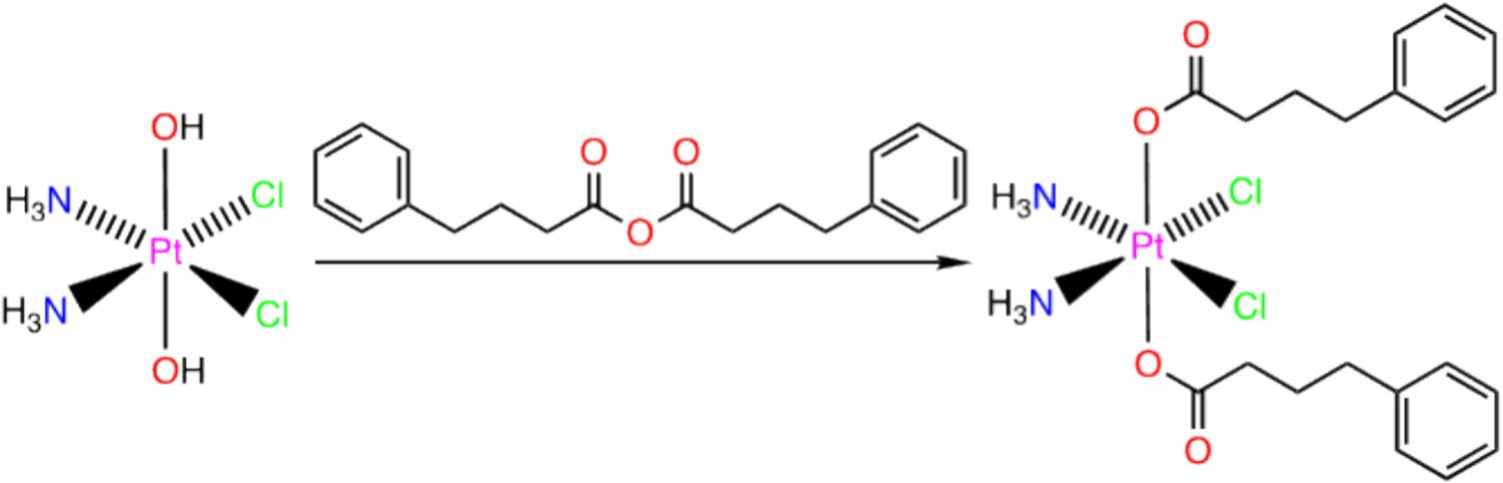
Synthesis schema of *cis*,* cis*,* trans*-[Pt(NH_3_)_2_Cl_2_(PBA)_2_] [Compound B] Addition of 4-phenylbutanoic anhydride reagent to *cis*,* cis*,* trans*-[Pt(NH_3_)_2_Cl_2_(OH)_2_] results in the substitution of PBA HDAC inhibitor ligands for the hydroxides on *cis*,* cis*,* trans*-[Pt(NH_3_)_2_Cl_2_(OH)_2_]. See Supplementary Fig. S1 and S2 for NMR and HR-ESI-MS validation

Compound B was prepared using modifications to a previously reported procedure (Fig. 1) [30]. To a stirring solution of 4-phenylbutanoic anhydride (244 mg, 0.748 mmol) in dimethyl formamide (DMF) (2 mL) was added *cis*,* cis*,* trans*-[Pt(NH_3_)_2_Cl_2_(OH)_2_] (100 mg, 0.298 mmol). The mixture was first heated at 40 °C for 2 h and then stirred at room temperature for 22 h. At the end of the stirring, all solids were fully dissolved. The DMF was removed under reduced pressure, and the remaining residue was resuspended in 3 mL of acetone and filtered after sonication. The filtrate was added dropwise to a stirring solution of diethyl ether (30 mL), precipitating the desired product as a white solid, which was isolated by vacuum filtration, washed with diethyl ether (3 × 10 mL), and dried *in vacuo*. Yield: 100 mg (0.160 mmol, 54%). ^1^H NMR (400 MHz, DMSO-*d*_6_): δ 7.29–7.15 (m, 10 H), 6.53 (br s, 6 H), 2.59 (t, *J* = 7.6 Hz, 4 H), 2.21 (t, *J* = 7.4 Hz, 4 H), 1.74 (quint, *J* = 7.5 Hz, 4 H). ESI-MS (negative ion mode): *m/z* 624.0998 ([M–H]^–^, calculated 624.0996). Analytical calculated for C_20_H_28_Cl_2_N_2_O_4_Pt: C, 38.35; H, 4.51; N, 4.47. Found: C, 38.62; H, 4.59; N, 4.48.

### Zebrafish Maintenance

Wild-type AB zebrafish (Cat. #: ZL1) were obtained from the Zebrafish International Resource Center (Eugene, OR). *wt1b*:eGFP zebrafish were obtained from Dr. Iain Drummond (MDI Biological Laboratory, Bar Harbor, ME) and *brn3c* (*pouf4f3*):eGFP fish were obtained from the University of Oregon, Eugene, OR. Zebrafish embryos were maintained in petri dishes with E3 water media (5 mM NaCl, 0.17 mM KCl, 0.33 mM CaCl_2_, 0.33 mM MgSO_4_, 1 ppm methylene blue (all Sigma Aldrich, St. Louis, MO)) at 28 °C with a 14-hour light and 10-hour dark cycle according to established protocols [31]. All zebrafish experiments were conducted in accordance with the guidelines of the Animal Care and Use Committee of the University of Mississippi Medical Center (UMMC), Jackson, MS, and approved by the UMMC Institutional Biosafety and IACUC Review Committees (IACUC protocol number: 2021 − 1161).

### Cell Culture

A2780 (cisplatin-sensitive; Cat.#: 93112519) and A2780cis (cisplatin-resistant; Cat.#: 93112517) ovarian cell lines were obtained from Sigma-Aldrich (St. Louis, MO) were cultured in RPMI-1640 media (Sigma) with 2 mM glutamine (Sigma), 10% fetal bovine serum (FBS, Gibco, Gaithersburg, MD) and 1:100 penicillin/streptomycin (Invitrogen, Carlsbad, CA) and incubated at 37 °C. To maintain cisplatin resistance, the A2780cis cell line was also treated with 1 µM cisplatin every 2–3 passages.

### Cell viability assay

The 3-(4,5-dimethylthiazol-2-yl)-2,5-diphenyltetrazolium bromide (MTT) assay was performed according to the procedure in [32]. Cells were seeded at 50,000 cells per well in 24-well plates and incubated for 24 h at 37 °C in 5% CO_2_. Then, wells were treated for 48 h in replicates of three in three separate experiments with a concentration series (500, 50, 5, 0.5, 0.05 µM) of cisplatin in media vehicle or compound B in 0.3% dimethyl sulfoxide (DMSO) vehicle. A negative control (media only), 0.3% DMSO vehicle control, positive control (Triton X-100), and set of blanks (media only with no cells) were also simultaneously performed in replicates of three in three separate experiments. The MTT assay was conducted for 2 h, with plates read using a spectrophotometer (BioTek Gen5, Winooski, VT) at 570 nm and 690 nm absorbance wavelengths. IC_50_ values were calculated in PRISM (GraphPad version 10, La Jolla, CA) using a sigmoidal, four parameter logistic equation. Standard deviation values were calculated using ED50 plus v1.0 online software (Mexico City, Mexico).

### Xenograft migration assay

2-day postfertilization (dpf) AB zebrafish embryos were dechorionized, anesthetized with 0.2 mg/mL tricaine methanesulfonate (MS-222) (Sigma), and placed into petri dishes with E3 water media. Ovarian cancer cell suspensions were collected from cell culture dishes using 0.025% trypsin (Gibco). Samples of 1 million cells/ml in cell culture media with 5 µl of Vybrant Dil, (Thermo Fisher Scientific, Waltham, MA) per mL of cell suspension were prepared and set aside on ice. Next, 100 K per µl of DiI labelled ovarian cancer cells were prepared in phosphate buffer saline (PBS; Invitrogen, Carlsbad, CA), and loaded into a borosilicate glass capillary needle (World Precision Instruments, Sarasota, FL) pulled by a micropipette puller (Narishige, PN-30, Amityville, NY). Loaded needles were then placed into a Narishige stereotaxic apparatus and connected to a Tritech Research microinjector (Los Angeles, CA) with the following settings: injection pressure, 10 kPa; holding pressure, 0 kPa; clear pressure, 10 kPa; and injection time, 0.1 s. Embryos were positioned on an agarose stage covered with E3 water placed under a Labomed Luxeo 6z stereo microscope (Fremont, CA) and were then injected with ~ 100–200 cells into their yolk sac. Embryos were then placed back into the incubator, and after 1-day post-injection (dpi), zebrafish xenografts with the same tumor sizes were randomly assigned to treatment and control groups. At 1 dpi, embryos were treated with either E3 media control, 0.3% DMSO/E3 solvent control, 0.9% NaCl/E3 solvent control, cisplatin in 0.9% NaCl/E3 solvent or Compound B in 0.3% DMSO/E3 solvent and were then placed back into the incubator. After 3 dpi, embryos were fixed in paraformaldehyde (Sigma) and kept in a 4 °C refrigerator overnight for imaging using an Axio Imager 2 (10X) and a Nikon Eclipse E600 (Tokyo, Japan). ImageJ (National Institutes of Health, MD) was used to calculate the corrected total cell fluorescence (CTCF) of samples using the formula: integrated density – (area of selected cell × mean fluorescence of background) [24].

### General toxicity assay

General toxicity was analyzed by quantifying apoptosis as previously described in [19, 20]. Therefore, 72 h post fertilization (hpf) AB embryos were treated for 48 h with an IC_50_ value of one of the platinum compounds or a control (0.9% sodium chloride (NaCl) [cisplatin control], 0.3% DMSO [compound B control], or 10 nM carbonyl cyanide m-chlorophenyl hydrazine [CCCP], a positive control). Then, embryos were anaesthetized using MS-222, fixed in 4% paraformaldehyde overnight at 4 °C, and transferred and stored in 100% methanol. Whole mount in situ hybridization followed by caspase-3 antibody (primary: ABCAM (Cambridge, UK); secondary: ThermoFisher Scientific) staining was performed on stored embryos. Images were taken using an Axio Imager 2 and processed with Adobe Photoshop software (Adobe Incorporated, San Jose, CA). For each experiment, 15 embryo sample sets were evaluated in three independent experiments, and two-way ANOVAs were performed with a Dunnett’s multiple comparison test with data presented as means ± SEM with *p* < 0.05 being significant.

### Ototoxicity assay

To quantify ototoxicity, the *brn3c* (*pouf4f3*):eGFP zebrafish transgenic, which expresses GFP in neuromast hair cells, was used [21]. Embryos were treated as described in the migration assay and were then placed into a 28ºC incubator for 48 h before analysis. Embryos were then anaesthetized with MS-222 and microscopically examined using an Axio Imager 2 (10X) and a Nikon Eclipse E600 (60X), and the number of hair cells in 10 randomly selected cranial and caudal neuromasts were counted and recorded. For each experiment, 15 embryo sample sets were evaluated in three independent experiments, and two-way ANOVAs were performed followed by a Fisher’s multiple comparison test with data presented as means ± SEM with *p* < 0.05 being significant.

### Nephrotoxicity assays

Analysis of treatment effect on renal area was performed using (*wt1b*):eGFP transgenic zebrafish that express GFP in the fused glomerulus and the pronephric tubules [15]. Embryos were treated with an IC_50_ value of either cisplatin, compound B, solvent vehicle negative control (0.9% NaCl [cisplatin control], or 0.3% DMSO [compound B control]) and were then placed into a 28ºC incubator for 48 h before analysis. Treated embryos were then anaesthetized with MS-222 and microscopically imaged using an Axio Imager 2. Renal area was measured using the Zeiss region-of-interest tool. Experiments were repeated in triplicate with 15 embryos per treatment group, and statistical analysis used two-way ANOVAs followed by a Fisher’s multiple comparison test with data presented as means ± SEM with *p* < 0.05 being significant.

Analysis of treatment effect on renal function was performed using AB wild-type zebrafish according to a procedure adapted from [14] that allows us to measure the clearance of rhodamine labelled dextran from a defined region of the zebrafish embryo circulatory system. Therefore, at 48 hpf, dechorionized zebrafish were anesthetized with 0.2 mg/mL tricaine methanesulfonate (MS-222) and embryos were positioned on an agarose stage covered with E3 water placed under an Olympus S2 microscope (Tokyo, Japan). Embryos were microinjected with 50 ng of rhodamine-labeled dextran (10 kDa, Invitrogen, Carlsbad, CA) into their sinus venosus using the same procedure, equipment and settings as in the xenograft migration assay. Drug and control treatment, microscopy and statistical analysis was the same as for the renal area experiments except that fluorescent measurements were made using ImageJ (National Institutes of Health, Bethesda, MD). CTCF) was calculated as in the xenograft migration assay.

## Results

MTT assays were performed to determine the IC_50_ values of cisplatin and compound B treatments in both A2780 cisplatin-sensitive and A2780cis cisplatin-resistant cell lines (Supplemental Table 1) for subsequent analytical steps. Cisplatin in the A2780 cell line had an IC_50_ value of 1.90 µM ± 0.91; whereas, compound B treatment had a value of 0.29 µM ± 0.31 (Supplemental Table 1). In the A2780cis cell line, cisplatin had an IC_50_ value of 15.74 µM ± 0.23, while compound B treatment had a value of 0.51 µM ± 0.12 (Supplemental Table 1). As these values are similar to those obtained in [6], we applied values of 2 or 15 µM cisplatin and 0.3 or 0.6 µM compound B in subsequent experiments.

**Fig. 2:**
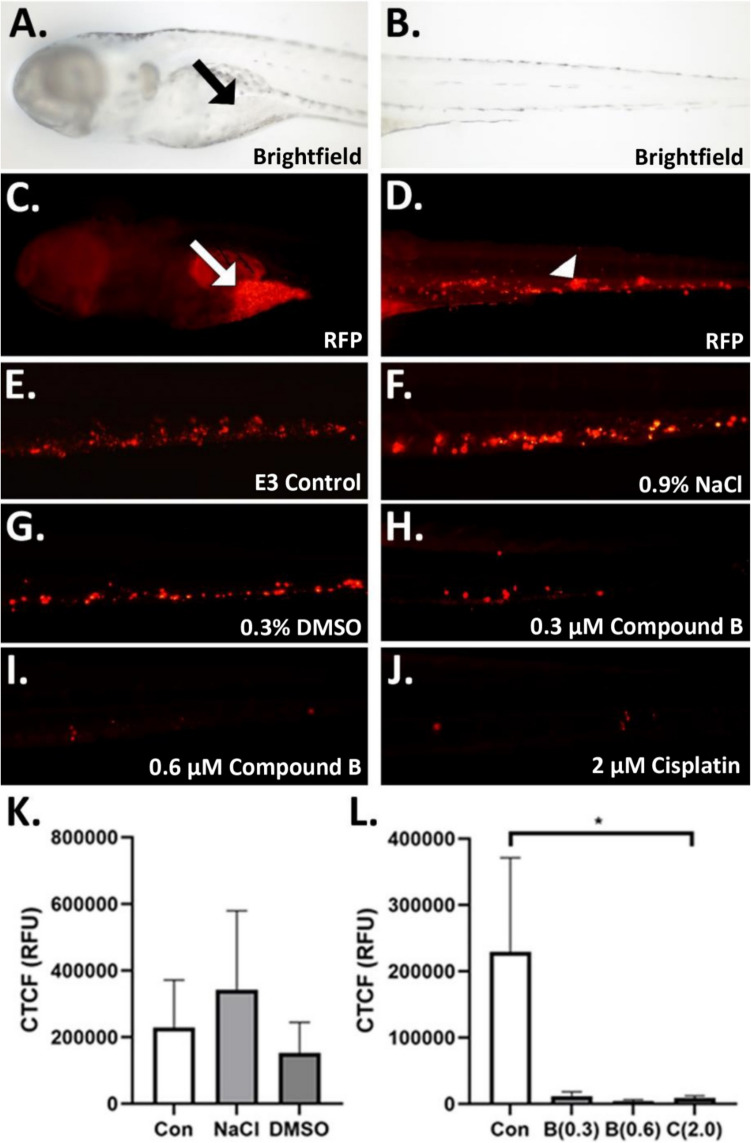
Compound B reduces ovarian cancer metastasis comparably to cisplatin.Images show ovarian cancer cells labeled with DiI membrane stain visible in the red fluorescent channel. **A.-D.** Representative images of microinjected zebrafish embryos (**A.** brightfield cranial view showing yolk sac injection site (black arrow); **B.** brightfield caudal view; **C.** red channel fluorescent cranial view showing yolk sac injection site with cancer cells (white arrow); **D.** red channel fluorescent caudal view showing metastasized A2780cis cells (white arrow head), **E.-J.** Representative images of control and experimental treatment zebrafish (**E.**, E3 media control; **F.**, 0.9% NaCl cisplatin control; **G.**, 0.3% DMSO compound B control; **H.**, 0.3 µM compound B; **I.**, 0.6 µM compound B; **J.**, 2.0 µM cisplatin). **K.** Comparison of E3 media (Con), 0.9% NaCl, and 0.3% DMSO controls. **L.** Comparison of E3 control (Con) with three platinum complex treatments: 0.3 µM compound B [B(0.3)], 0.6 µM compound B [B(0.6)], 2.0 µM cisplatin [C(2.0)]. CTCF = corrected total cell fluorescence; *p* < 0.05, “*” = 0.05; *N* = 5–15

Then, we determined the effect of the different control and drug treatments on cisplatin-resistant ovarian cancer metastasis using AB wild-type zebrafish that were microinjected with A2780cis cells (Fig. 2A-J). We found no difference between the control treatment mean values: E3 media, 229,081 RFU; NaCl, 342,575 RFU; DMSO, 152,436 RFU (Fig. 2K). However, when we compared the E3 control with the experimental treatments, we found that both concentrations of compound B (0.3 and 0.6 µM) caused a similar reduction in metastasis as did cisplatin at 2.0 µM (mean RFU values: E3 media, 229081; 0.3 µM compound B, 11782; 0.6 µM compound B, 4553; 2.0 µM cisplatin, 8961; Fig. 2L) confirming the efficacy of compound B to be at a lower concentration that cisplatin *in vivo*.

**Fig. 3:**
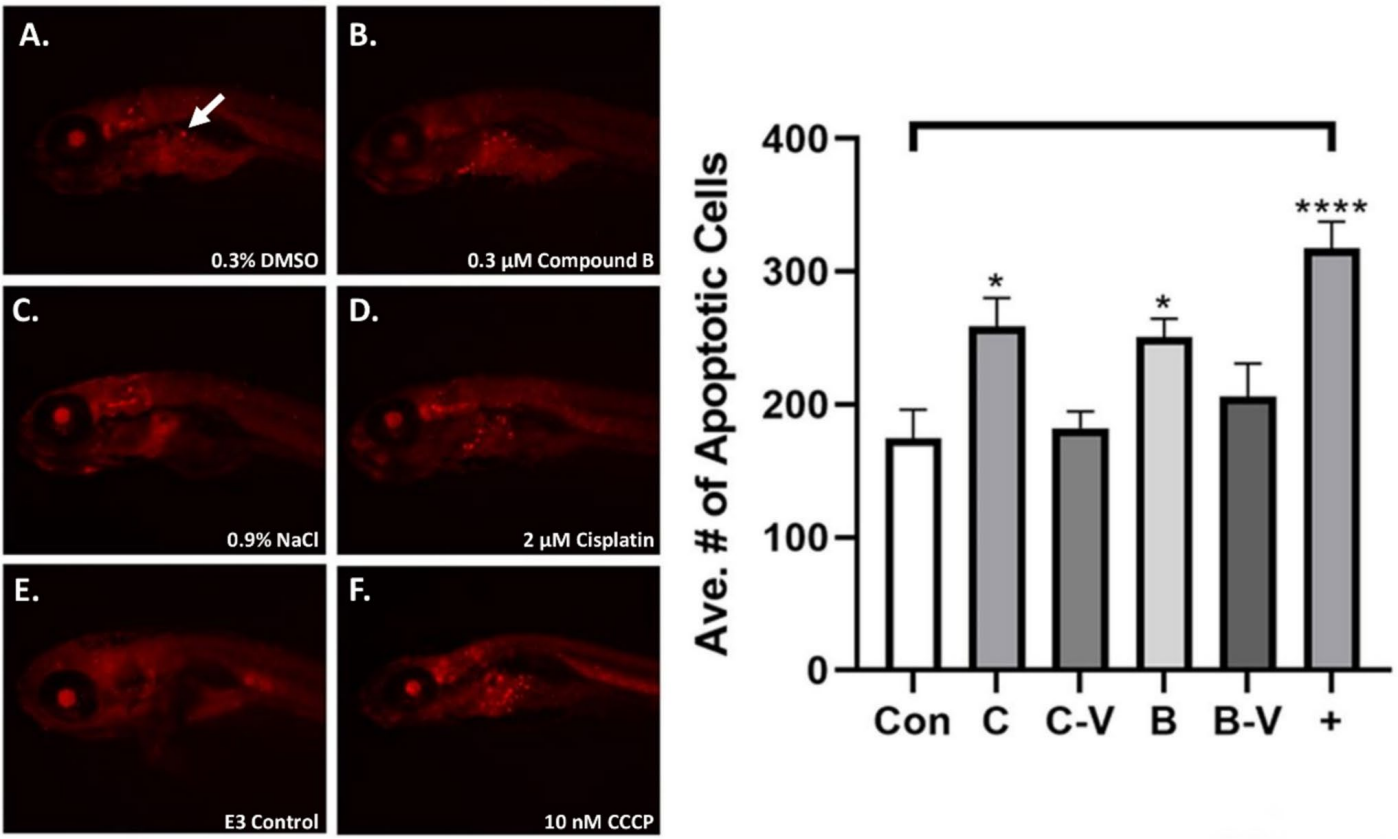
Cisplatin and compound B treatment caused increased general tissue cell apoptosis in zebrafish AB embryos A.-F. Representative images of control and experimental compound (**A.**, 0.3% DMSO; **B.**, 0.3 µM compound B; **C.**, 0.9% NaCl; **D.**, 2 µM cisplatin; **E.**, E3 media; **F.**, 10 nM CCCP) treated zebrafish embryos). **G.** Graph of average number of apoptotic cells observed for control and experimental compound treated zebrafish embryos. Arrow in **A.** indicates caspase-3 positive cell. Key: Con = E3 egg water media control; C = cisplatin; C-V = cisplatin 0.9% NaCl vehicle control; B = compound B; B-V = 0.3% DMSO compound B vehicle; CCCP = 10 nM carbonyl cyanide m-chlorophenyl hydrazine positive control; *p* < 0.05, “*” = 0.05, “****” = 0.0001

Next, we determined the effect of control (E3, 0.3% DMSO, 0.9% NaCl [negative controls], and 10 nM carbonyl cyanide m-chlorophenyl hydrazone (CCCP) [a positive control used to induce cell death in zebrafish] [33]) and drug (0.3 µM compound B and 2 µM cisplatin) treatments on general zebrafish embryo toxicity by exposing 72 hpf AB wild-type embryos to treatments for 48 h and measured the number of cells expressing cleaved-caspase 3 in the whole embryo (Fig. 3A-F). We found the following mean numbers of apoptotic cells: E3, 174.5; 0.9% NaCl, 182.3; 2 µM cisplatin, 258.6; 0.3% DMSO, 250.5; 0.3 µM compound B, 206.0; 10 nM CCCP, 317.6, with cisplatin and compound B causing similar levels of apoptotic cells, and CCCP positive control treatment causing the highest level of general tissue toxicity (Fig. 3G).

**Fig. 4:**
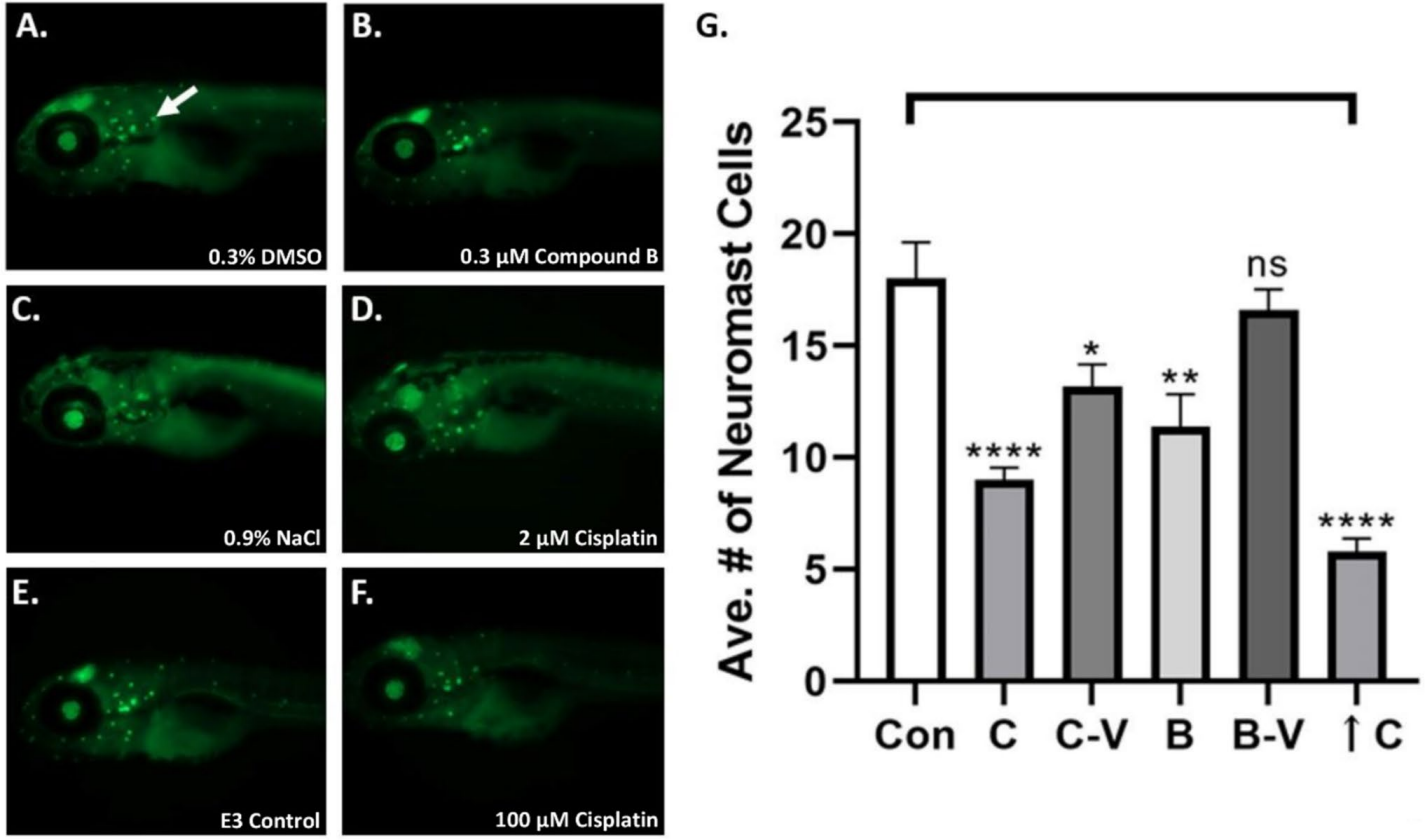
Cisplatin treatment decreased neuromast hair cell numbers more than compound B in *brn3c*(*pouf4f3*):eGFP zebrafish A.-F. Representative images of control and experimental compound (**A.**, 0.3% DMSO; **B.**, 0.3 µM compound B; **C.**, 0.9% NaCl; **D.**, 2 µM cisplatin; **E.**, E3 media; **F.**, 100 µM cisplatin) treated *brn3c* (*pouf4f3*):eGFP zebrafish embryos. **G.** Graph of average number of neuromast hair cells counted for control and experimental compound treated zebrafish embryos. Arrow in **A.** indicates neuromast expressing GFP reporter. Key: Con = E3 egg water media control; C = cisplatin; C-V = cisplatin 0.9% NaCl vehicle control; B = compound B; B-V = 0.3% DMSO compound B vehicle control; ↑C = 100 µM cisplatin overdose used as a positive control; *p* < 0.05, “*” = 0.05, “**” = 0.01, “****” = 0.0001, ns = non-significant

To assess ototoxicity, we used the *brn3c* (*pouf4f3*):eGFP zebrafish transgenic, and applied the same treatments as in the general toxicity assay (except an overdose of 100 µM cisplatin was substituted as a positive control to target auditory hair cells without inducing mortality in the embryos), and then measured the number of GFP expressing hair cells in zebrafish neuromasts (Fig. 4A-F). We found the following mean numbers of hair cells in the samples: E3, 18.0; 0.9% NaCl, 13.2; 2 µM cisplatin, 9.0; 0.3% DMSO, 16.6; 0.3 µM compound B, 11.4; 100 µM cisplatin, 5.8, with cisplatin, NaCl cisplatin vehicle, compound B, and 100 µM cisplatin treatments causing decreased numbers of hair cells compared to E3 media control (order: 100 µM cisplatin < 2 µM cisplatin < 0.3 µM compound B < NaCl cisplatin vehicle; Fig. 4G).

**Fig. 5:**
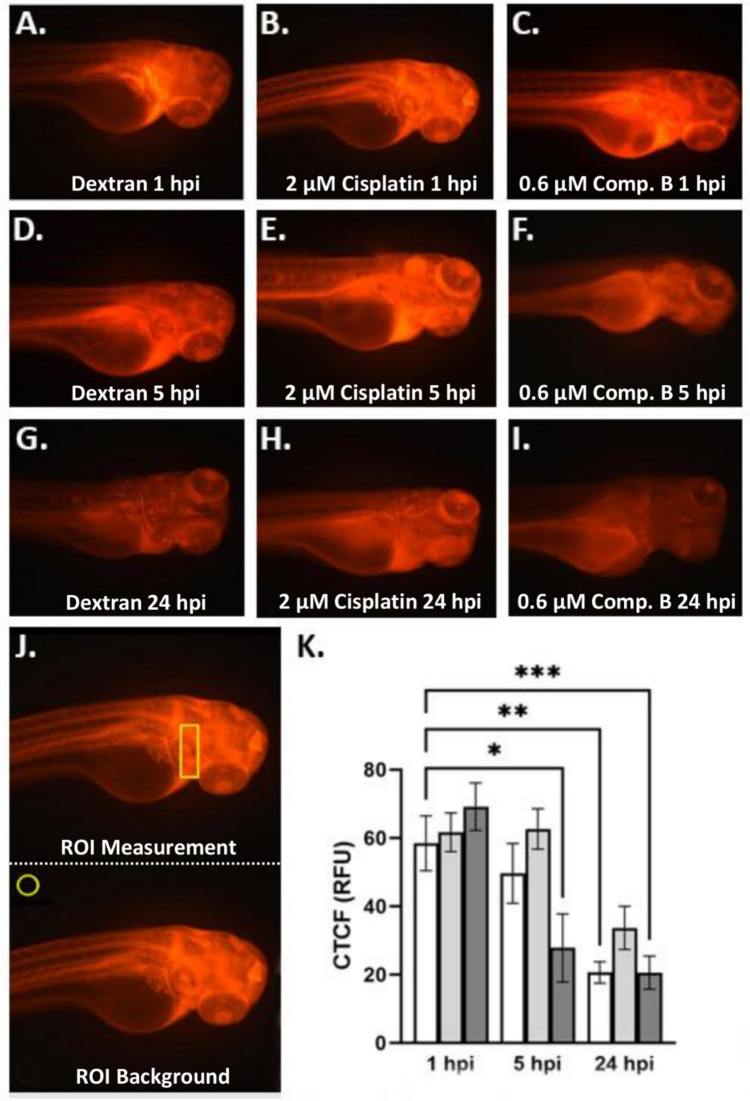
Compound B does not cause decreased glomerular filtration in AB zebrafish A.-I. Representative images of control and experimental compound treated AB zebrafish embryos showing red fluorescent dye distribution at 1, 5, and 24 hpi (**A.**, dextran control-1 hpi; **B.**, 2 µM cisplatin-1 hpi; **C.**, 0.6 µM compound B-1 hpi; **D.**, dextran control-5 hpi; **E.**, 2 µM cisplatin-5 hpi; **F.**, 0.6 µM compound B-5 hpi; **G.**, dextran control-24 hpi; **H.**, 2 µM cisplatin-24 hpi; **I.**, 0.6 µM compound B-24 hpi; ), **J.** Examples of region-of-interest (ROI) analysis (top panel: representative polygon for ROI measurement of fluorescence; bottom panel: representative polygon for ROI measurement of background), **K.**, Graph of fluorescent intensity for control and experimental compound treated zebrafish embryos at 1, 5, and 24 hpi. Key: CTCF (RFU) = corrected total cell fluorescence in relative fluorescence units; white columns = dextran control; light gray columns = 2 µM cisplatin; dark gray columns = 0.6 µM compound B; *p* < 0.05, “*” = 0.05, “**” = 0.01, “***” = 0.001; *N* = 5–10

In order to assess nephrotoxicity, we initially evaluated whether the treatments used in the ototoxicity assay altered the size of the zebrafish embryonic pronephros in (*wt1b*):eGFP transgenic zebrafish that express GFP in the fused glomerulus and the pronephric tubules. However, we found that the treatments did not alter mean renal structure size compared to the E3 control (E3, 5004 µm^2^; 0.9% NaCl, 5112 µm^2^; 2 µM cisplatin, 4880 µm^2^; 0.3% DMSO, 5229 µm^2^; 0.3 µM compound B, 4742 µm^2^; 100 µM cisplatin, 4700 µm^2^; Supplemental Fig. S3). Therefore, we substituted a renal function test in AB zebrafish to assess whether any of the treatments impaired kidney filtration. We measured the presence of dextran conjugated red fluorescence dye in embryo samples at 1, 5, and 24 h post injection (hpi) (Fig. 5A-I) and then used ImageJ to calculate CTCF values from representative areas and background measurements (Fig. 5J). We found that only E3 control and compound B treatment was associated with decreased dye retention after 24 hpi when compared to 1 hpi control samples (E3 control at 1 hpi mean = 58.44 RFU; E3 control at 24 hpi mean = 20.63 RFU; compound B at 24 hpi mean = 20.59 RFU) (Fig. 5K).

## Discussion

A chemotherapeutic that potentiates the effect of cisplatin in cancer cell culture might have different effects against cancer *in vivo* and may promote toxicity in non-cancerous cells. For example, the platinum(II) complex, phenanthriplatin, provides superior anti-cancer efficacy than cisplatin in many cancer cell lines, but it has only an equivalent effect against cancer tumor volume in mouse xenograft models [34] and can cause more severe side-effects than cisplatin [32]. Further, the platinum(IV) pro-drug compound B has demonstrated synergistic anticancer behavior between its cisplatin-core and PBA HDAC inhibitor ligands in cisplatin-sensitive and -resistant ovarian cancer cell lines [6, 35]. Compound B also causes increased platination of nuclear DNA, increased reactive oxygen species levels and apoptosis in these ovarian cell lines [6], which are molecular effects associated with cisplatin-mediated cell death in non-cancerous tissue [1] and cisplatin associated side-effects, e.g., nephrotoxicity and ototoxicity [3]. To better understand how compound B acts *in vivo* against ovarian cancer and general organismal toxicity and nephro- and ototoxicity, we synthesized and validated this platinum(IV) complex according to standard procedures [6, 28–30] (Fig. 1; Supplemental Fig. S1 and S2). Then, we used the MTT assay [32] to measure the effect of cisplatin and compound B in cisplatin-sensitive and -resistant ovarian cancer cell lines [6, 35] (Supplemental Table 1). This data suggests that a lower dosage of compound B (0.3 µM) than cisplatin (2.0 µM) can counteract the effect of cisplatin resistance in ovarian cancer cells and might act as a functional dosage to comparatively measure compound B and cisplatin in zebrafish models.

Combinatorial administration of cisplatin and HDAC inhibitors can reduce ovarian cancer metastasis in cell culture models [36], but the effects of their combination on cancer cell migration *in vivo* is not currently well understood. We first comparatively evaluated the controls used in the metastasis assay as NaCl levels may alter zebrafish osmoregulation and effect physiology [37], and DMSO has been shown to modulate differentiation in ovarian cancer culture models and proliferation and inflammatory responses in other cultured cell types [38, 39]. However, the addition of NaCl or DMSO to E3 media did not change cancer metastasis in the samples (Fig. 2A-C, G) suggesting that the solvent controls do not contain chemical agents which can modulate biological function differently than E3 media alone. We also found that the IC_50_ values of compound B (0.3 µM) and cisplatin (2.0 µM) decreased metastasis to a similar level (Fig. 2D, F, H). As this concentration of compound B is nearly 10-fold lower than that of cisplatin, this data suggests that inclusion of the PBA HDAC inhibitor may be able to potentiate the effect of cisplatin to promote DNA damage by promoting nucleic acid binding [6] or act on oncogenes and/or tumor suppressors as reported for some HDAC inhibitors [8–11] to prevent metastasis. This interpretation is further supported by the data for a higher concentration (0.6 µM) of compound B, where increasing compound B treatment produced a downward trend in the amount of metastasized ovarian cancer cells (Fig. 2E, H). These results suggest that the combination of cisplatin and PBA may synergize to prevent metastasis at lower concentration than cisplatin alone potentially by PBA modulation of histone deacetylase regulation of DNA platination or cancer gene function.

The combination of cisplatin with PBA could cause different *in vivo* cell death effects than that from their separate administration. Both cisplatin and HDAC inhibitors can modulate cell death mechanisms, e.g., caspases, in cancer cell lines [40, 41], but the action of the platinum(II) drug and PBA together to activate apoptotic pathways in non-cancerous tissue has not been previously analyzed. As HDAC inhibition should promote greater cisplatin targeting of DNA via increasing double helix exposure to the platinum-based drug [6], simultaneous introduction of PBA with cisplatin could promote generalized tissue toxicity *in vivo*. Interestingly, we found that cisplatin and compound B caused a similar increase in the number of cells undergoing apoptosis (Fig. 3). Further, the distribution of caspase-3 positive cells does not appear to be focused in any specific region of the embryos (Fig. 3A-F) suggesting that cisplatin and compound B may have similar pharmacokinetic properties. Also, as PBA is a reversible inhibitor of Class I and II HDACs [42] that are highly conserved between zebrafish and mammals with the exception of the class I HDAC, HDAC2, which zebrafish lack [43], it is likely that the zebrafish model should replicate the effects of general organismal toxicity from PBA treatment in humans. It is also uncertain whether the level of general organismal toxicity observed in these experiments represents a qualitative, e.g., physiological effect *in vivo*.

The general toxicity assay did not allow assessment of the effect of cisplatin or compound B on specialized renal and auditory cell types that are often damaged during cisplatin treatment [3]. Because compound B treatment caused increased cisplatin-mediated platination of DNA and reduced cell viability in cancer cell lines [6], it is possible that similar effects could occur in specialized renal and auditory cells leading to more severe side-effects. The ototoxicity data images (Fig. 4A-F) showed that compound B treatment at 0.3 µM caused less hair cell loss compared to 2.0 µM cisplatin (Fig. 4G). We also noticed that 0.9% NaCl cisplatin vehicle caused a reduction in hair cell counts (Fig. 4G). This result suggests that NaCl may also have an effect alone or together with cisplatin to damage the externally presented neuromast hair cells of zebrafish, possibly by regulating osmolarity in these sensory cells [44]. Interestingly, when we measured the area of the zebrafish pronephron and linked glomerulus after control or experimental treatment (Supplemental Fig. S3A-F), we did not find any effect on renal structure (Supplemental Fig. S3G). This outcome suggests that developmental biology mechanisms may supervene and prevent treatment-modulated effects as the zebrafish kidney undergoes continuous formation during the time-frame of these experiments (72–120 hpf) [45]. However, the renal function assay data (Fig. 5A-F) showed that cisplatin treatment (2.0 µM), but not vehicle or a higher dosage of compound B (0.6 µM), decreased dye filtration (Fig. 5G). Both the ototoxicity and nephrotoxicity function assays suggest that administering a reduced concentration of cisplatin as in compound B at 0.3 µM or 0.6 µM, may act to minimize cisplatin-mediated DNA damage to auditory hair or renal cells. An alternative interpretation could be that PBA-mediated HDAC inhibition might promote the function of genes associated with DNA repair, but this interpretation is not supported in cancer cell studies where HDAC inhibition typically promotes DNA damage and prevents the action of DNA repair mechanisms [46, 47].

In conclusion, platinum(IV) complexes with cisplatin-cores and HDAC inhibitor axial ligands have been proposed as anti-cancer drugs due to their ability to mitigate cisplatin resistance in cell lines. Here, we demonstrated that the platinum(IV) complex, compound B, which has a cisplatin-core and two phenylbutyrate HDAC inhibitor axial ligands, not only causes reduced cell viability in ovarian cancer cell lines that are cisplatin-sensitive and -resistant, but at lower dosages than cisplatin, has comparable *in vivo* efficacy to reduce cisplatin-resistant ovarian cancer cell metastasis and general organismal toxicity. We also showed that compound B treatment caused reduced *in vivo* levels of damage to auditory cells and renal function compared to cisplatin. This data suggests that platinum(IV) complexes which act to maximize cisplatin targeting of DNA through prevention of histone acetylation may represent a promising future avenue for chemotherapeutic development.

## Supplementary Information

Below is the link to the electronic supplementary material.

ESM1 (DOCX 561 KB)

## Data Availability

All data supporting the findings of this study are available within the paper and its Supplementary Information.
