# Supplementary material for: Anti-ovarian cancer migration and toxicity characteristics of a platinum(IV) pro-drug with axial HDAC inhibitor ligands in zebrafish models
Source: Invest New Drugs. 2024 Oct 21;42(6):644–54. doi: 10.1007/s10637-024-01479-3 (PMC11625067; doi:10.1007/s10637-024-01479-3)
Supplement: Supplementary file 1 — (DOCX 561 KB) [file 10637_2024_1479_MOESM1_ESM.docx]

**Article Title:** Anti-ovarian cancer migration and toxicity characteristics of a platinum(IV) pro-drug with axial HDAC inhibitor ligands in zebrafish models

**Journal Name:** Investigational New Drugs

**Author Names:** Salma Begum, Scheldon D. Irvin, Carol K. Cox, Zhouyang Huang, Justin J. Wilson, Jerry D. Monroe, Yann Gibert

Corresponding author: Yann Gibert, Department of Cell and Molecular Biology, University of Mississippi Medical Center, 2500 State Street, Jackson, MS, 39216, email: ygibert@umc.edu

**Supplemental Table**

**Table 1. IC_50_ values for cisplatin and compound B in A2780 and A2780cis cell lines.**

| **Treatment** | **IC_50_ (µM)** |
| --- | --- |
| Cisplatin (A2780) | 1.90 ± 0.91 |
| Compound B (A2780) | 0.29 ± 0.31 |
| Cisplatin (A2780cis) | 15.74 ± 0.23 |
| Compound B (A2780cis) | 0.51 ± 0.12 |

**Supplemental Figures**

**
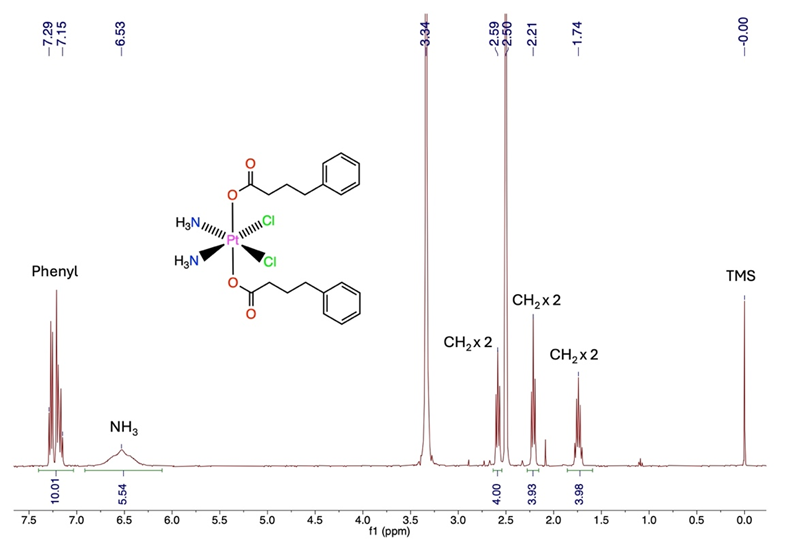
**

**Fig. S1** **^1^H NMR spectrum of *cis,cis,trans*-[Pt(NH_3_)_2_Cl_2_(PBA_2_)] in DMSO-*d*_6_ (400 MHz, 25 °C)**

**
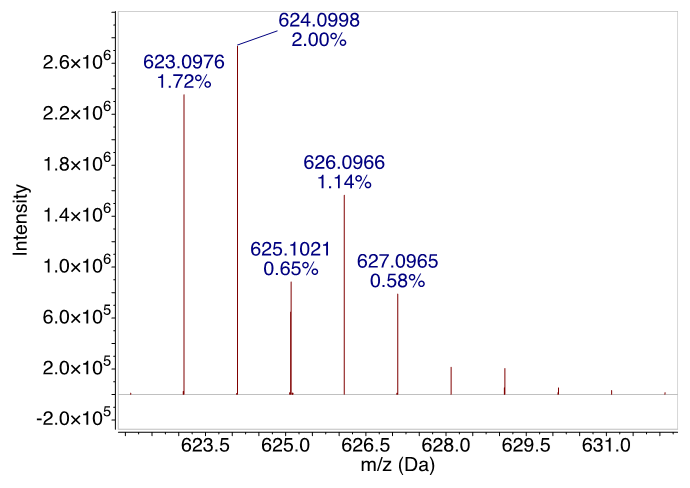
**

**Fig. S2** **HR-ESI-MS of *cis,cis,trans*-[Pt(NH_3_)_2_Cl_2_(PBA_2_)]**


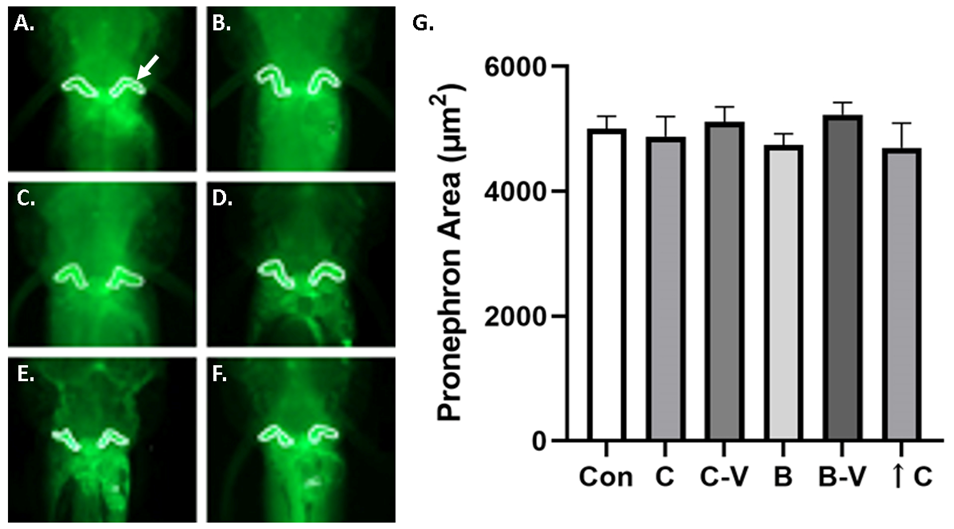


**Fig. S3 Cisplatin and compound B do not alter pronephron area in *wt1b*:eGFP zebrafish A.-F.** Representative images of control and experimental compound treated (**A.**, 0.3% DMSO; **B.**, 0.3 µM compound B; **C.**, 0.9% NaCl; **D.**, 2 µM cisplatin; **E.**, E3 media; **F.**, 100 µM cisplatin) *wt1b*:eGFP zebrafish embryos showing green fluorescent pronephrons with white tracing around the perimeter of the pronephron structures. **G.** Graph of pronephron area observed for control and experimental compound treated zebrafish embryos. Arrow in **A.** indicates pronephron with border tracing. Key: Con = E3 egg water media control; C = cisplatin; C-V = cisplatin 0.9% NaCl vehicle control; B = compound B; B-V = 0.3% DMSO compound B vehicle control; ↑C = 100 µM cisplatin overdose used as a positive control; *p* < 0.05.
